# COVID-19 Shock and the Time-Varying Volatility Spillovers Among the Energy and Precious Metals Markets: Evidence From A DCC-GARCH-CONNECTEDNESS Approach

**DOI:** 10.3389/fpubh.2022.906969

**Published:** 2022-07-27

**Authors:** Xiaoyu Tan, Xuetong Wang, Shiqun Ma, Zhimeng Wang, Yang Zhao, Lijin Xiang

**Affiliations:** ^1^School of Finance, Zhongnan University of Economics and Law, Wuhan, China; ^2^School of Finance, Shandong University of Finance and Economics, Jinan, China

**Keywords:** energy market, precious metal market, DCC-GARCH-CONNECTEDNESS, volatility spillover, COVID-19

## Abstract

The outbreak of the COVID-19 epidemic intensified the volatility of commodity markets (the energy and precious metals markets), which created a significant negative impact on the volatility spillovers among these markets. It may also have triggered a new volatility risk contagion. In this paper, we introduce the DCC-GARCH-CONNECTEDNESS approach to explore the volatility spillover level and multi-level spillover structure characteristics among the commodity markets before and during the COVID-19 epidemic in order to clarify the new volatility risk contagion patterns across the markets. The results implied several conclusions. (i) The COVID-19 epidemic has significantly improved the total volatility spillover level of the energy and precious metals markets and has enhanced the risk connectivity among the markets. (ii) The COVID-19 epidemic has amplified the volatility of the crude oil market, making it the main volatility spillover market, namely the source of volatility risk contagion. (iii) The COVID-19 epidemic outbreak enhanced the external risk absorption capacity of the natural gas and silver markets, and the absorption level of the external volatility spillover improved significantly. Furthermore, the risk absorption capacity of the gold market weakened, while the gold market has remained the endpoint of external volatility risk during the epidemic and has acted as a risk stabilizer. (iv) The volatility spillover among markets has clear time-varying characteristics and a positive connectedness with the severity of the COVID-19 epidemic. As the severity of the COVID-19 epidemic increases, the volatility risk connectivity among the markets rapidly increases.

## Introduction

The COVID-19 epidemic has spread rapidly around the world since the beginning of 2020, affecting the global economy and deeply and dramatically impacting global commodity markets. As the benchmark of global commodities, the energy and precious metals markets have suffered a particularly prominent loss.

The spread of COVID-19 has caused the international energy market, which was involved in the U.S.-Iraq conflict, to experience a new round of disturbances. These have mainly presented through a sharp decline in industrial and commercial energy demand due to the COVID-19 outbreak. The isolation measures for continued epidemic spread have further reduced fuel demand in the transportation industry. At the same time, the supply-side producers have not taken collective action to restrict production and ensure price. COVID-19 has created a both-way instability between energy supply and demand, and its consequences have been particularly prevalent in the international crude oil and gas markets. As a commodity with dual economic and political attributes, the economic and strategic significance of crude oil is undeniable. In statistics and empirical studies, international crude oil prices have also been confirmed to closely relate to the global economy (1). The collapse of oil demand caused by the COVID-19 epidemic has been considered the most serious turbulence since energy became a global commodity. In addition, the global natural gas market, which is undergoing structural adjustment, has faced additional severe pressure. On the one hand, the previously excessive supply directly affected natural gas due to the insufficient demand caused by the epidemic. On the other hand, the decline in crude oil prices also indirectly weakened the economy of associated gas. The pressure from the epidemic and the downward side effects in other markets have led to a decline in the price of natural gas, with the price even falling to near the marginal cost of production. The COVID-19 epidemic, though, has made the global natural gas industry more sensitive to supply and demand. In combination with the indirect impact of the crude oil sector (2), the industry is in serious distress.

COVID-19 has directly impacted the supply and demand of the global capital market, and its igniting of investor panic should be considered. COVID-19 has influenced the precious metals market, characterized by the dual attributes of goods and finance, to attract a large amount of capital. This is striking when compared to the decline in global risk asset prices. As traditional hedging commodities, gold and silver are fully representative of the panic-driven turmoil in precious metals markets. As a recognized hard currency in precious metals, gold is a reserve asset during periods of economic stability and a hedging tool during periods of economic anomalies. In other words, it is sensitive to the impact of external emergencies (3). During the spread of the epidemic, the international gold price rose. The rise was driven by global panic, but the decline of other commodity prices limited its ability to rise further. Due to multiple effects, gold price behaved sensitively, exhibiting a volatile pattern. Although its risk aversion function is slightly inferior to that of gold, the silver market is also located in the risk exposure category under the epidemic because of its important role in industry. Existing research fully confirms the positive role of silver (4). The spread of the COVID-19 epidemic has suppressed consumer demand from downstream of the industry, further impacting silver prices through global industrial chain channels. This shows that, in multiple channels, silver has also been considerably sensitive to COVID-19. Sensitive volatility in crude oil, natural gas, gold, and silver prices is also reasonably representative in theory and statistics during periods of economic turmoil or downturn. They are sensitive to market trends and epidemic outbreaks and can highlight the impact of investor sentiment (5). This sensitivity can in turn lead to inevitable risk spillover. On the one hand, the closely association between energy products means there must be a complex relationship among the price of crude oil and its products. On the other, consensus has been reached that energy commodities can impact other markets (6, 7). In particular, fossil energy represented by crude oil impacts the price of precious metals by affecting the production cost of the metal industry (8). Under the background of the both-way supply and demand obstruction caused by the epidemic and related panic, then, research into the volatility spillover connections within and between the two key commodity markets is more valuable than ever.

This paper applies a DCC-GARCH-CONNECTEDNESS approach employed by Gabauer (9) and Karim et al. (10) to capture the impact of the COVID-19 epidemic on major commodity markets for energy and precious metals. We further explore the directional dynamic connectedness between the energy and precious metals markets, which are represented by the crude oil, natural gas, gold, and silver price compound return sequence, respectively. The results showed that, under the COVID-19 epidemic, the volatility spillover connections—namely risk contagion between the energy and precious metals markets—intensified from a dynamic perspective, and the volatility spillover level positively correlated with the severity of the epidemic. The outbreak of COVID-19 made the crude oil market a source of risk spillover. At the same time, the natural gas and silver markets' capacity to absorb external risks has increased significantly. The gold market's ability to avoid risks has declined but still plays a key role in risk hedging.

This study makes several contributions to the existing literature. Given the complexity of the COVID-19 epidemic and its impact, we applied a DCC-GARCH-CONNECTEDNESS approach to describe the time-varying volatility spillover between the energy and precious metals markets from a dynamic perspective. The DCC-GARCH-CONNECTEDNESS approach combines the dynamic conditional correlation-generalized autoregressive conditional heteroscedasticity (DCC-GARCH) model, the volatility impulse response functions (VIRF), and the connectedness approach of Diebold and Yilmaz (11). Furthermore, by satisfying the GARCH effect feature of commodity price volatility, this approach can overcome the lack of dynamic directional description of risk connections through traditional methods, owning the advantage of remaining independent of the variable ordering.

The remainder of this paper is organized as follows. Literature review reviews the literature pertaining to the shock of the COVID-19 epidemic on commodity markets. Methodology and data introduces the methodology applied in this paper and describes the data. Empirical results and disscussion presents the empirical results, and conclusion concludes the paper.

## Literature Review

Research into COVID-19's impact on global markets has burgeoned. Regardless of the theoretical or empirical point of view taken, most scholars' results have shown that the COVID-19 epidemic will increase volatility in global markets and strengthen the risk spillover effect among markets (12, 13). While it is worth noting that the current research focuses on the volatility of the securities market under COVID-19, such as in the classical stock (14–16) and bond markets (17, 18), and the research on the risk spillover effects of commodity markets under the background of the outbreak of COVID-19 epidemic also take capital market as one of the main research objects. Examples include the interactive impact between the stock and crude oil markets during the epidemic (19, 20) and the volatility connections between precious metals and other capital assets (21, 22). In addition, some scholars only study the commodity markets volatility during the period of COVID-19 epidemic, but the sample market is relatively single, such as energy markets (23–25) or gold markets (26), which is difficult to clarify the structural characteristics of commodity risk spillover.

Research has made some progress in assessing COVID-19's impact on the commodity market. However, it has an increasingly sensitive position in the international economy to connect the commodity and financial markets. Consequently, the academic community's focus on the commodity market remains insufficient, especially compared to the traditional capital market. The existing literature largely focuses on the link between the stock and commodity markets under the COVID-19 epidemic or only the single commodity market. Studies have paid less attention to the dynamic relationships among different markets within the commodity market. However, as an energy-intensive industry, the precious metals market is sensitive to volatility in the energy market (27). The epidemic's heterogeneous effects on energy consumption in different industries, such as crude oil and natural gas, have also been confirmed (28). The phenomenon of internal risk resonance and the subsequent dislocation it causes deserves increased attention (29). Considering the energy and precious metals markets' complex relationship and key position in the wake of COVID-19, this paper considers the dynamic connections between them as the research object. This perspective makes up for the deficiency of existing literature in the research perspective.

In terms of research methods, existing studies on the risk spillover between financial markets under the COVID-19 epidemic have largely applied the VAR family model, mainly including TVPVAR (29, 30), PVAR (19), and others. The Diebold and Yilmaz (11, 31, 32) volatility spillover measure has also been widely used (33–35). Some scholars have introduced the network to describe the risk spillover connections among financial markets (36, 37), and GARCH and its extended models have also been adopted to some extent (7, 38, 39).

In summary, the research methods used to depict financial market volatility caused by external shocks have been correspondingly solid. Based on the classical VAR and GARCH models, scholars have expanded the models according to their research directions, using the Diebold and Yilmaz (11, 31, 32) method, the VECM model, and network density as their common assistant methods. Considering the volatility clustering and leverage effect of crude oil price (40, 41), applying GARCH family models can better describe and capture the volatility characteristics. In addition, the change in international, political, and economic circumstances has made the financial attributes of commodities increasingly prominent, showing more complex time-varying and dynamic characteristics. The energy and precious metals markets heavily participate in the international economy through production and consumption channels, and the volatility of their return rate is a prominent manifestation in the age of COVID-19. The existing literature on and the research methods used to explore this complex and dynamic connection leave room for further expansion. To that end, this paper introduces the DCC-GARCH-CONNECTEDNESS approach, which fully considers the characteristics of the research object—namely return rate's volatility—and meets the needs of dynamic identification under the shadow of COVID-19.

Based on the above discussion, this paper aims to solve the following problems: (1) What is the dynamic impact of the COVID-19 epidemic on the energy and precious metals markets? (2) What is the level and status of risk spillovers among the energy and precious metals markets under the impact of the COVID-19 epidemic? (3) What dynamic volatility spillover connections and risk transfer paths between energy and precious metals markets were caused by the COVID-19 epidemic? This paper selects crude oil, natural gas, gold, and silver as the representative industries of the two markets and constructs a DCC-GARCH-CONNECTEDNESS approach to examine the volatility spillover of their price returns and to estimate the impact of the COVID-19 epidemic. Furthermore, we analyze the changes in directional dynamic connectedness within commodity markets. Our research expands the existing literature, enriches research on the volatility spillover characteristics of the commodity market under the spread of the COVID-19, and accounts for the deficiency of research perspectives and methods.

## Methodology and Data

### Methodology

The time-varying volatility spillover indexes were constructed using the following three aspects of the DCC-GARCH-CONNECTEDNESS approach: (1) time-varying total connectedness, (2) time-varying directional connectedness, and (3) time-varying net pairwise directional connectedness. The specific construction process of various volatility spillover indexes was as follows:

First, the DCC-GARCH model was constructed. The two-step DCC-GARCH model employed by Engle (42) was utilized to examine time-varying conditional volatility. Specifically, the DCC-GARCH (1,1) model was written as follows:


(1) $$\begin{array}{r} y_{t}=\mu_{t}+\varepsilon_{t}\varepsilon_{t}|F_{t-1}\sim \;N\left(0,H_{t}\right) \end{array}$$

(2) $$\begin{array}{r} \varepsilon =\sqrt{H_{t}}u_{t}u_{t}\sim N\left(0,I\right) \end{array}$$

(3) $$\begin{array}{r} H_{t}=D_{t}R_{t}D_{t} \end{array}$$

where *F*_*t*−1_ represents all available information up to *t*−1. *y*_*t*_, μ_*t*_, ϵ_*t*_, and *u*_*t*_ are *N* × 1-dimensional vectors that represent the analyzed time series, conditional mean, errors, and standardized errors, respectively. Moreover, *R*_*t*_, and $D_{t}=diag\left(\sqrt{h_{11t}},\cdots \;,\sqrt{h_{NNt}}\right)$ were *N* × *N*-dimensional matrices that represented the dynamic conditional correlations, time-varying conditional variance-covariance matrices, and time-varying conditional variances, respectively.

First, *D*_*t*_was constructed using the GARCH model constructed by Bollerslev (43) to estimate each sequence. Based on Hansen and Lunde's (44) study, one shock and one persistency parameter were assumed:


(4) $$\begin{array}{r} h_{ii,t}=\varpi +\alpha \varepsilon_{i,t-1}^{2}+\beta h_{ii,t-1} \end{array}$$

The dynamic conditional correlations were calculated as follows:


(5) $$R_{t}=diag\left(1/\sqrt{q_{ii,t}},\ldots ,1/\sqrt{q_{NN,t}}\right)Q_{t}diag\left(1/\sqrt{q_{ii,t}},\ldots ,1/\sqrt{q_{NN,t}}\right)$$

(6) $$\begin{array}{r} Q_{t}=\left(1-a-b\right)\overset{-}{Q}+au_{t-1}u_{t-1}^{{}^{\prime }}+bQ_{t-1} \end{array}$$

where *Q*_*t*_ and $\overset{-}{Q}$ are *N* × *N*-dimensional positive-definite matrices that illustrate variance-covariance matrices of conditional and unconditional standardized residuals, respectively, and where *a*(α) and *b*(β) are non-negative shock and persistency parameters that satisfy the *a*+*b* < 1(α+β ≤ 1) condition. If condition *a*+*b* < 1 is fulfilled, *Q*_*t*_ and *R*_*t*_ will vary over time. Alternatively, the model will converge to the CCC-GARCH model, where *R*_*t*_ does not change with time.

Subsequently, we calculated the volatility impulse response functions (VIRF), which has the same meaning as the generalized impulse response functions (GIRFs) introduced by Koop et al. (45) and Pesaran and Shin (46), owning the advantage of remaining independent of the variable ordering. This method can be interpreted as the J-step-ahead impact of a shock in one variable on another variable's conditional volatilities, which can be written as


(7) $$\begin{array}{l} \psi^{v}=VIRF(J,\delta_{j,t},F_{t-1})=E(H_{t+J}|\varepsilon_{j,t},F_{t-1})-E(H_{t+J}|\varepsilon_{j,t}\\ \;=0,F_{t-1}) \end{array}$$

where δ_*j, t*_ represents the selection vector with one on the *jth* position and zero otherwise.

It is worth noting that forecasting the conditional variance-covariances utilizing the DCC-GARCH model is the heart version of the VIRF and can be accomplished iteratively in the three steps.

First, GARCH (1,1) was used to predict conditional volatility (*D*_*t*+*h*_|*F*_*t*_). The specific process was as follows:


(8) $$\begin{array}{r} E\left(h_{ii,t+1}|F_{t}\right)=\varpi +\alpha \delta_{1,t}^{2}+\beta h_{ii,t}h=1 \end{array}$$

(9) $$\begin{array}{r} E\left(h_{ii,t+h}|F_{t}\right)=\overset{h-1}{\underset{i=0}{\sum }}\varpi {\left(\alpha +\beta \right)}^{i}+{\left(\alpha +\beta \right)}^{h-1}\\ \;E\left(h_{ii,t+h-1}|F_{t}\right)h>1 \end{array}$$

Second, *E*(*D*_*t*+*h*_|*F*_*t*_) could be predicted:


(10) $$\begin{array}{r} E\left(Q_{t+1}|F_{t}\right)=\left(1-a-b\right)\overset{-}{Q}+au_{t}u_{t}^{{}^{\prime }}+bQ_{t}h=1 \end{array}$$

(11) $$\begin{array}{r} E\left(Q_{t+h}|F_{t}\right)=\left(1-a-b\right)\overset{-}{Q}+aE\left(u_{t+h-1}u_{t+h-1}^{{}^{\prime }}|F_{t}\right)\\ \;+bE\left(Q_{t+h-1}|F_{t}\right)h=1 \end{array}$$

where$E\left(u_{t+h-1}u_{t+h-1}^{{}^{\prime }}|F_{t}\right)\approx E\left(Q_{t+h-1}|F_{t}\right)$ (42), which helped to predict the dynamic conditional correlations.

Third, the conditional variance-covariances could be expressed as


(12) $$\begin{array}{r} E\left(R_{t+h}|F_{t}\right)\approx diag\left[E\left(\sqrt{q_{ii,t+h}}|F_{t}\right),\ldots ,E\left(\sqrt{q_{NN,t+h}}|F_{t}\right)\right]\\ E\left(Q_{t+h}\right)diag\left[E\left(\sqrt{q_{ii,t+h}}|F_{t}\right),\ldots ,E\left(\sqrt{q_{NN,t+h}}|F_{t}\right)\right] \end{array}$$

(13) $$\begin{array}{r} E\left(H_{t+h}|F_{t}\right)\approx E\left(D_{t+h}|F_{t}\right)E\left(R_{t+h}|F_{t}\right)E\left(D_{t+h}|F_{t}\right) \end{array}$$

Finally, the generalized forecast error variance decomposition (GFEVD), which can be interpreted as the variance share one market has on others, was calculated based on the VIRF. These shares were then normalized so that each row summed up to one, meaning that all markets together explained 100% of the forecast error variance of market *i*. This was calculated as follows:


(14) $$\begin{array}{r} {\tilde{\phi }}_{ij,t}^{g}\left(J\right)=\frac{\sum_{t=1}^{J-1}\psi_{ij,t}^{2,v}}{\sum_{j=1}^{N}\sum_{t=1}^{J-1}\psi_{ij,t}^{2,v}} \end{array}$$

where, $\sum_{j=1}^{N}\phi_{ij,t}^{g}\left(J\right)=1$ and $\sum_{i,j=1}^{N}\phi_{ij,t}^{g}\left(J\right)=N$.

Thus, the total connectedness index (TCI) could be constructed as


(15) $$\begin{array}{r} TCI_{t}^{g}=\frac{\sum_{i,j=1,i\neq j}^{N}{\tilde{\phi }}_{ij,t}^{g}\left(J\right)}{N} \end{array}$$

Simultaneously, we constructed the time-varying pairwise connectedness index as well, which calculated the mean level $\left(P_{i\to j,t}^{g}\left(J\right)\right)$ of volatility spillover from market *i* to market *j* and the mean level ($P_{i\leftarrow j,t}^{g}\left(J\right)$) of volatility spillover from market *j* to market *i*. The indexes are as follows:


(16) $$\begin{array}{r} P_{i\to j,t}^{g}\left(J\right)=\frac{\overset{g}{\underset{ji,t}{\phi }}\left(J\right)}{\sum_{j=1}^{N}\overset{g}{\underset{ji,t}{\phi }}\left(J\right)}*100 \end{array}$$

(17) $$\begin{array}{r} P_{i\leftarrow j,t}^{g}\left(J\right)=\frac{\overset{g}{\underset{ij,t}{\phi }}\left(J\right)}{\sum_{i=1}^{N}\overset{g}{\underset{ij,t}{\phi }}\left(J\right)}*100 \end{array}$$

We extracted Formulas (16) and (17) and defined the net pairwise volatility spillover among the energy and precious metals markets as formulas (16) minus formulas (17). This was calculated as


(18) $$\begin{array}{r} P_{i,t}^{g}=P_{i\to j,t}^{g}\left(J\right)-P_{i\leftarrow j,t}^{g}\left(J\right). \end{array}$$

Subsequently, we constructed the time-varying directional connectedness index and calculated the volatility spillover from market to all other markets ( volatility spillover) and the volatility spillover index from all other markets to market *i* (*FROM*_*it*_ volatility spillover). The expressions are as follows:


(19) $$\begin{array}{r} TO_{it}=\sum_{j=1,j\neq i}^{N}P_{i\to j,t}^{g}\left(J\right) \end{array}$$

(20) $$\begin{array}{r} FROM_{it}=\sum_{i=1,j\neq i}^{N}P_{i\leftarrow j,t}^{g}\left(J\right). \end{array}$$

Subtracting formulas (19) and formulas (20) from each other led to the net total directional connectedness:


(21) $$\begin{array}{r} NET_{it}=TO_{it}-FROM_{it} \end{array}$$

If *NET*_*it*_ is positive (negative), it means that market is a net transmitter (receiver) of volatility risk.

### Data

In this paper, we represent precious metals markets through the gold and silver markets and represent energy markets through the crude oil and natural gas markets. Four active foreign exchange trading funds (ETFS) were selected to represent the gold, silver, crude oil, and natural gas markets; in other words, we recorded the closing price of the fund as the asset price and then used the logarithmic difference of the fund's closing price to represent the compound return of this kind of asset. Doing so reflected the market performance, providing real and reasonable data information for the fluctuation correlation analysis among commodity markets. The specific selection of funds is shown in Table 1. The trading data of the exchange traded funds (ETFs) comes from *investment.com* (https://cn.investing.com/indices/).

**Table 1:** The selection of exchange traded funds (ETFs).

**ETFs**	**Market**	**Tracking target**
The US Oil fund ETF (USO)	Crude oil market	The prices of WTI crude oil
The US natural gas fund ETF (UNG)	Natural gas market	The prices of natural gas
SPDR gold shares trust ETF (GLD)	Gold market	The price of gold bullion in the over-the-counter(OTC) market
iShares silver trust ETF (SLV)	Silver market	The price of the silver

*The trading data of the exchange traded funds (ETFs) comes from investment.com (https://cn.investing.com/indices/)*.

To clarify COVID-19's impact on the volatility spillover connectedness between commodity markets—in other words, the market risk contagion structure—the sample intervals selected in this paper included the periods before and during the COVID-19 epidemic. In addition, considering the availability of sample data and the comparability of the estimated results of the sub-samples, the sub-samples selected in this paper were drawn between October 18, 2017 and January 22, 2020 and between January 23, 2020 to March 18, 2022, respectively. We used the start date of the COVID-19 epidemic data published by Johns Hopkins University as the virus's outbreak date. The sample data was the daily frequency information. The two sample intervals contained 568 trading days, and the full sample had a total of 4,544 daily frequency data. The descriptive statistics of each variable are shown in Table 2.

**Table 2:** Description statistics.

**Variables**	**Observations**	**Mean**	**Std.dev**	**Min**	**Max**	**Skewness**	**Kurtosis**
***Pre- COVID-19***							
*Slv*	568	0.00003	0.00480	−0.02260	0.01835	−0.30016	5.50205
*Gld*	568	0.00014	0.00289	−0.01055	0.01102	0.10003	4.24630
*Oil*	568	0.00010	0.00808	−0.03229	0.04980	−0.08215	6.59926
*Gas*	568	−0.00041	0.01116	−0.09219	0.07518	−0.12934	16.46450
***During COVID-19***							
*Slv*	568	0.00025	0.00959	−0.06345	0.03742	−0.78707	10.37086
*Gld*	568	0.00015	0.00455	−0.02397	0.02058	−0.58685	6.75196
*Oil*	568	−0.00019	0.01527	−0.12677	0.06695	−2.43313	22.64316
*Gas*	568	0.00011	0.01530	−0.05102	0.06803	0.09068	4.34677

*Slv, Gld, Oil and Gas represent silver price compound return, gold price compound return, crude oil price compound return and natural gas price compound return respectively, which reflect silver market, gold market, crude oil market and natural gas market respectively, and the following is the same*.

In addition, the sample data used in this paper were time series data, and the stability test of each sequence data was an important prerequisite to ensure the accuracy and rationality of the estimation results. Consequently, the ADF unit root test was performed on the price compound return sequences of energy and precious metals before and during the COVID-19 epidemic. Moreover, to ensure the robustness of the test results, a PP test was also applied to verify the results of the ADF unit root test, as shown in Table 3. All kinds of asset return data sequences were found to be stationary sequences.

**Table 3:** The results of unit root tests.

	**ADF test**			**PP test**		
**Variables**	**Test statistic**	**1%Critical value**	**5%Critical value**	**PP test**	**1%Critical value**	**5%Critical value**
**Pre- COVID-19**						
*Slv*	−7.067^***^	−3.430	−2.860	−24.769^***^	−3.430	−2.860
*Gld*	−6.080^***^	−3.430	−2.860	−25.020^***^	−3.430	−2.860
*Oil*	−6.566^***^	−3.430	−2.860	−24.509^***^	−3.430	−2.860
*Gas*	−6.044^***^	−3.430	−2.860	−25.706^***^	−3.430	−2.860
**During COVID-19**						
*Slv*	−5.856^***^	−3.430	−2.860	−22.902^***^	−3.430	−2.860
*Gld*	−6.194^***^	−3.430	−2.860	−21.953^***^	−3.430	−2.860
*Oil*	−6.726^***^	−3.430	−2.860	−21.286^***^	−3.430	−2.860
*Gas*	−7.017^***^	−3.430	−2.860	−23.714^***^	−3.430	−2.860

*** *Denote the significance levels of 1%, the maximum lag order of ADF test is 12*.

## Empirical Results and Discussion

### Static Analysis of the Volatility Spillover of Commodity Price Compound Return Sequences

Based on the VIRF and GFEVD, this paper calculated the volatility spillover connectedness among the energy and precious metals markets before and during the epidemic, as shown in Table 4. The total volatility spillover level of the price compound return between markets before and during the COVID-19 epidemic was found to be 82.55 and 86.36%, respectively, and the average spillover rate of a single market was 20.64 and 21.59%, respectively. This indicates a significant volatility spillover connectedness among commodity markets.

**Table 4:** The volatility spillover of commodity price compound return sequences.

	***Slv***	***Gld***	***Oil***	***Gas***	***FROM***
***Pre-COVID-19 period***					
*Slv*	67.42	31.12	1.36	0.1	32.58
*Gld*	46.86	52.88	0.06	0.2	47.12
*Oil*	1.07	0.03	97.52	1.37	2.48
*Gas*	0.02	0.03	0.33	99.62	0.38
*TO*	47.95	31.18	1.75	1.67	82.55
*NET*	15.37	−15.94	−0.73	1.29	20.64
***During COVID-19 period***					
*Slv*	58.52	35.58	5.74	0.16	41.48
*Gld*	37.79	61.24	0.96	0.01	38.76
*Oil*	2.19	0.34	97.05	0.41	2.95
*Gas*	0.41	0.03	2.73	96.83	3.17
*TO*	40.4	35.95	9.43	0.58	86.36
*NET*	−1.08	−2.81	6.48	−2.59	21.59

*The data in the table is the static spillover level among energy and precious metal markets, and the results are based on the DCC-GARCH-CONNECTEDNESS approach*.

The outbreak of the COVID-19 epidemic also increased the closeness of volatility spillover connectedness among the gold, silver, crude oil, and natural gas markets and enhanced risk connectedness among commodity markets. It is worth noting as well that, before the COVID-19 epidemic broke out, silver was the main volatility spillover market with strong risk contagion capacity. In turn, the gold market was the main receiver of the external volatility spillover, and the risk transfer of the precious metal market was significantly stronger than that of the energy market. During the COVID-19 epidemic, the volatility spillover level of the silver market decreased rapidly, and the precious metals markets showed their risk absorption capacities under the shock of major emergencies which verified that the precious metals market could play the role of risk shock absorber in the case of increasing external economic uncertainty. The crude oil market became the main spillover of risk.

### Time-Varying Analysis of the Volatility Spillovers of Commodity Price Compound Return Sequences

**Figure 1:**
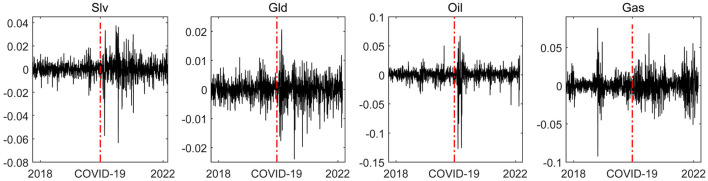
Volatility of compound return sequences of commodity prices. Black solid line represents the dynamic change of compound returns of commodity prices; the red dash line represents the starting point of the COVID-19 epidemic, and the starting date of the COVID-19 epidemic data at Johns Hopkins University is used as the outbreak date of the COVID-19 epidemic in this paper.

The COVID-19 epidemic has placed enormous pressure on global economic growth, creating a negative impact on financial systems that cannot be ignored. As Figure 1 shows, it has resulted in sharp surges in the volatility of the financial (12) and commodity markets. The red dash line represents the outbreak point of COVID-19. After the COVID-19 epidemic broke out, the stability of the price compound returns of silver, gold, crude oil, and natural gas rapidly declined and the volatility of the price return increased significantly. Therefore, it is of great practical significance to explore COVID-19's impact on the commodity market.

In addition, it is worth noting that the volatility of the commodity price compound return had time-varying characteristics and showed clear differences over time.

From the dynamic perspective, we conducted an in-depth study on the volatility spillover connectedness structure and its changes among the markets before and during the COVID-19 epidemic. We aimed to rectify the dynamic changes of the total volatility spillover level, the level of volatility spillover between the commodity markets, and the risk spillover status of the energy and precious metal markets. In so doing, we attempted to identify the spillover path and tendency of commodity markets' volatility risk under the continuous impact of the COVID-19 epidemic. We further clarified the COVID-19 epidemic's dynamic impact on the energy and precious metals markets in order to provide theoretical support and decision-making reference for the formulation of the COVID-19 epidemic control policies and the establishment of risk prevention and control mechanisms.

#### Time-Varying Total Connectedness

**Figure 2:**
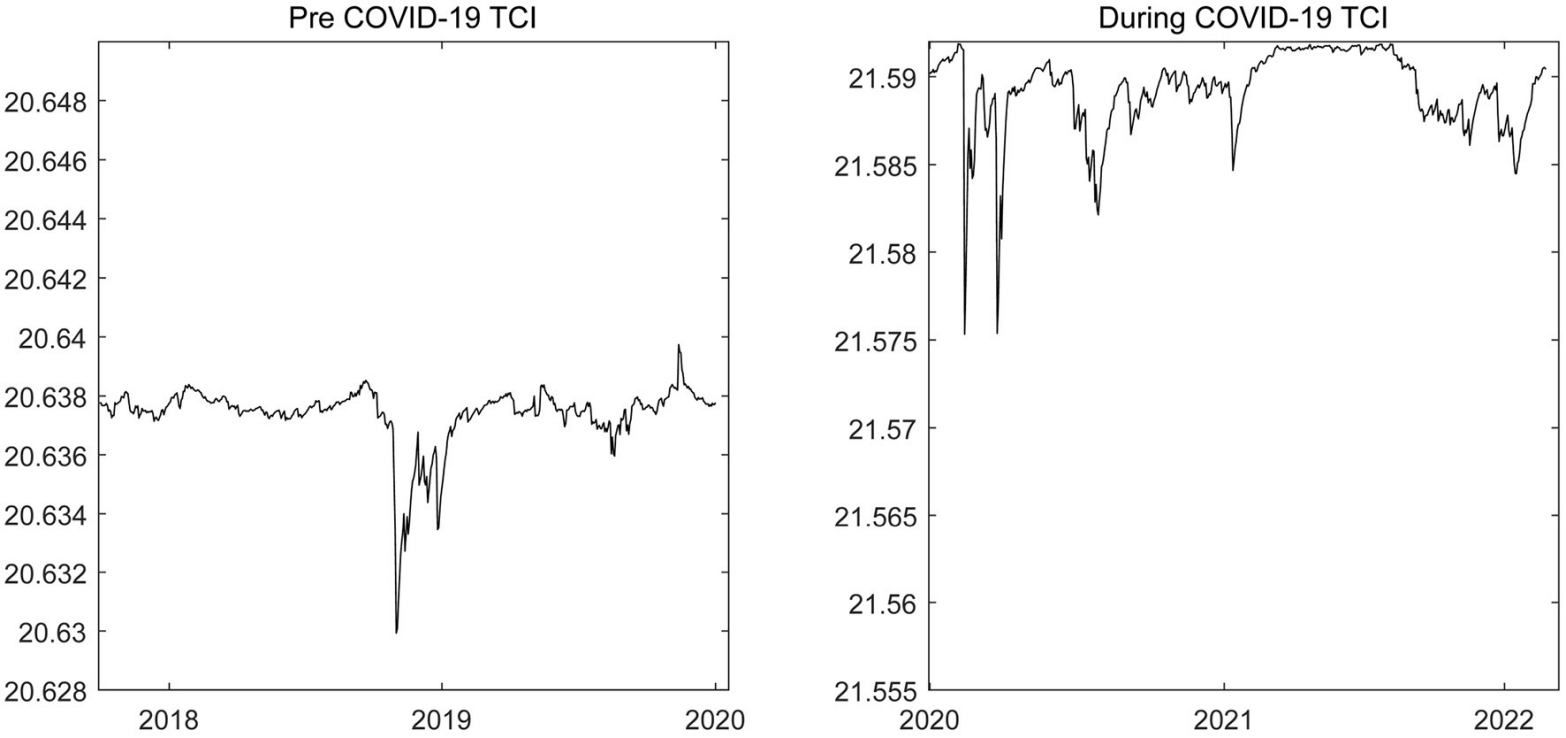
The time-varying total connectedness among energy and precious metals markets. The results are based on the DCC-GARCH-CONNECTEDNESS approach, and the solid line represents the change trend of the total volatility spillover effects of energy and precious metals markets.

The dynamic changes in the total volatility spillover level among the energy and precious metals markets before and during the COVID-19 epidemic are shown in Figure 2. During the period of the COVID-19 epidemic, the magnitude of the volatility spillover clearly increased, indicating that the COVID-19 epidemic has significantly affected the connectedness among the four types of commodity markets and has enhanced risk connectivity among markets. This finding confirms the conclusion of the static analysis and is consistent with the conclusions of Benlagha and Omari (47) and Farid et al. (35). The main reason may relate to the sentiment of the investors in markets (12, 48), which will amplify the negative impact of the COVID-19 epidemic shock.

Additionally, Figure 2 depicts that the impacts of the COVID-19 epidemic on the energy and precious metals markets had clear time-varying characteristics. For example, at the beginning of the epidemic, volatility spillover among the four commodity markets rapidly rose to a relatively high level. This primarily occurred because the global spread of the COVID-19 epidemic increased economic uncertainty and investor panic, leading to a rapid sell-off in global financial markets, including commodity markets. The rapid withdrawal of capital created a domino effect, causing the uncertainty qualitative risk to spread rapidly across the markets. This spread made the volatility spillover level of commodity markets rise rapidly in the early stages of the COVID-19 epidemic. With continuous improvements to epidemic prevention and control in the world, the level of market risk infection has declined periodically. However, the cross-border contagion of COVID-19 gives the epidemic the characteristics of strong repeatability and frequency (49). During 2020 and 2021, for instance, the volatility spillover level among the gold, silver, crude oil, and natural gas markets fluctuated greatly, showing a strong connectedness with the severity of the epidemic. This pattern was especially present in the early and middle portions of 2021 and early 2022. For example, on 23 April 2021, there were more than 1 million new confirmed cases per day in the world. As the global epidemic situation intensified, the level of volatility spillover among commodity markets increased rapidly as well. At the beginning of 2022, global epidemics resumed due to vaccine boycotts and rallies in some countries, and market volatility spillover continued to rise, as Figure 2 shows.

#### From and to Connectedness

**Figure 3:**
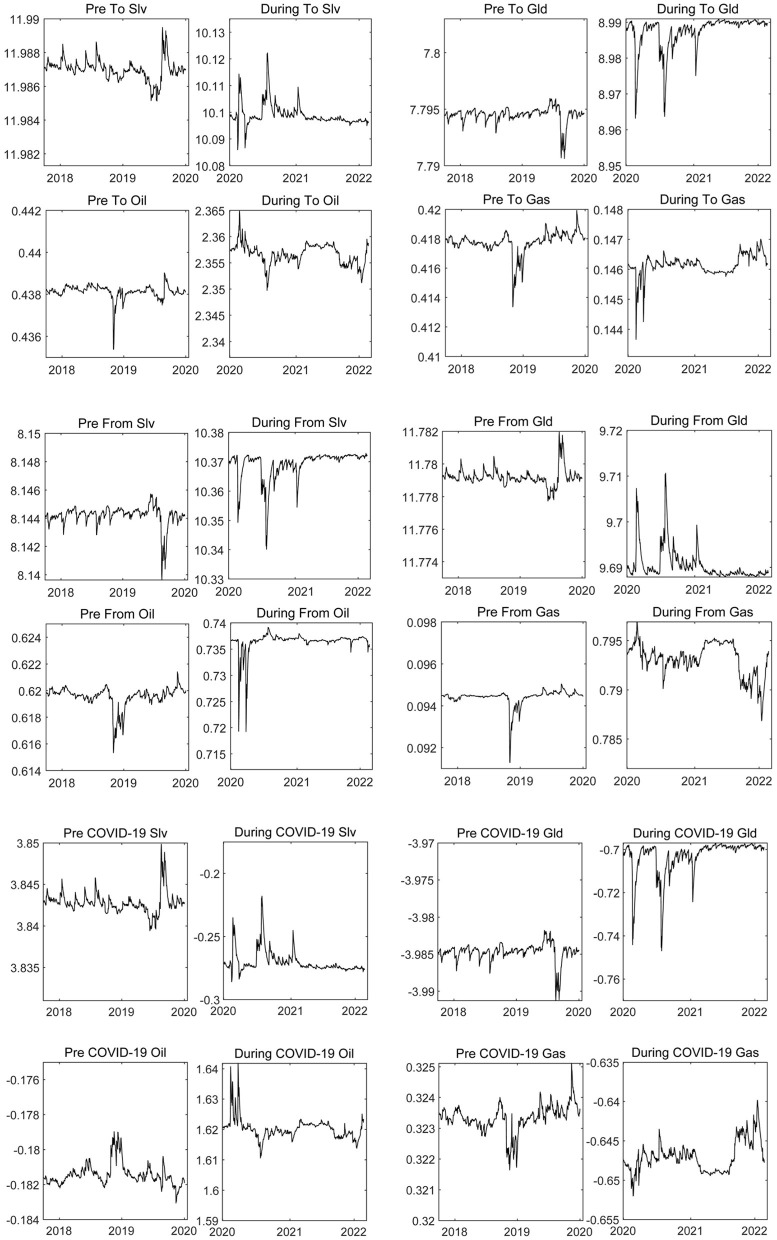
The time-varying directional connectedness of the energy and precious metals markets. The results are based on the DCC-GARCH-CONNECTEDNESS approach, and the solid line represents the change trend of the “TO volatility spillover,” “FROM volatility spillover” and “NET volatility spillover” of energy and precious metals markets; “Pre” indicates the period before the COVID-19 epidemic, and “During” indicates the COVID-19 epidemic period.

The COVID-19 epidemic has made significant heterogeneous impacts on the energy and precious metals markets and has significantly changed their volatility spillover ability and risk spillover status. This is exemplified in Figure 3.

The TO volatility spillover level of the silver market was found to have decreased, and the FROM volatility spillover level of the silver market was found to have increased. The volatility spillover level of the natural gas market also had the same characteristics, meaning the NET volatility spillover level of the silver and natural gas markets transitioned from positive to negative. We found that the silver and natural gas markets have become receivers of external volatility spillover under the COVID-19 epidemic. Furthermore, their risk absorption capacity has improved significantly, which mainly relates to the demands of economic recovery and excess earnings opportunities in certain countries. For example, under the COVID-19 epidemic, countries have sought the optimal path to recover their economies. They have preferred to accelerate economic transformation and replace the old kinetic energy driven by resources with the new kinetic energy brought by technological innovation. New energy technologies, such as 5G and solar energy, belong to silver-intensive fields, which enhance the anti-risk value of silver. Simultaneously, manufacturers' production reduction strategy during COVID-19 has been effective, reducing the price sensitivity of natural gas and increasing investment income. Consequently, its risk absorption capacity improved. Under the shock of the epidemic, the magnitude of TO volatility spillover and FROM volatility spillover of crude oil market has increased simultaneously, while the hoist scope of the TO volatility spillover level was decidedly higher than the FROM volatility spillover. Thus, the level of the crude oil market's NET volatility spillover has risen rapidly throughout the COVID-19 epidemic. The crude oil market changed from the receiver of the external volatility risk to the spillover of the volatility risk and became the source of risk contagion among commodity markets in the post-epidemic era. This may have occurred because the COVID-19 epidemic cut production supply chains around the world and weakened market demand expectations for oil, which undoubtedly exacerbated the volatility of international oil prices and improved its risk spillover levels. It is noteworthy that during the COVID-19 epidemic, the TO and FROM volatility spillover levels of the gold market showed reverse changes, and the net volatility spillover level of the gold market improved. This reveals that the negative impact of the COVID-19 epidemic weakened the risk absorption capacity of the gold market and mainly related to the enhanced risk reception capacity of the silver and natural gas markets. However, we found that the net volatility spillover level of the gold market before and during the COVID-19 epidemic was below the 0-scale line, and its absorption capacity of external volatility spillover was the strongest. This proves that gold with the property of hedging continues to stably play the role of risk damper, which is consistent with the research conclusion of Junttila et al. (50). Overall, the crude oil market is a source of risk under the shock of major emergencies, while the precious metals market can be used as an important tool to hedge the risk of financial asset price volatility. This conclusion was also supported by Mensi et al. (51).

Additionally, the spillover ability and status of commodity markets showed clear time-varying characteristics. As the COVID-19 epidemic increases in severity, the volatility spillover level of each market will enlarge. For example, in the early stages of the outbreak in 2020, the net volatility spillover levels of the silver, gold, and natural gas markets decreased significantly. Furthermore, all showed strong risk reception ability, whereas the level of risk spillover in the crude oil market rose rapidly. As countries paid more attention to the prevention and control of the epidemic, investors' preference gradually decreased, the frequency of capital flow decreased, and the stability of capital increased. Thus, the level of risk spillover and risk absorption of each market decreased. This point was mutually corroborated with the conclusions of our previous study.

#### Net Pairwise Directional Connectedness

**Figure 4:**
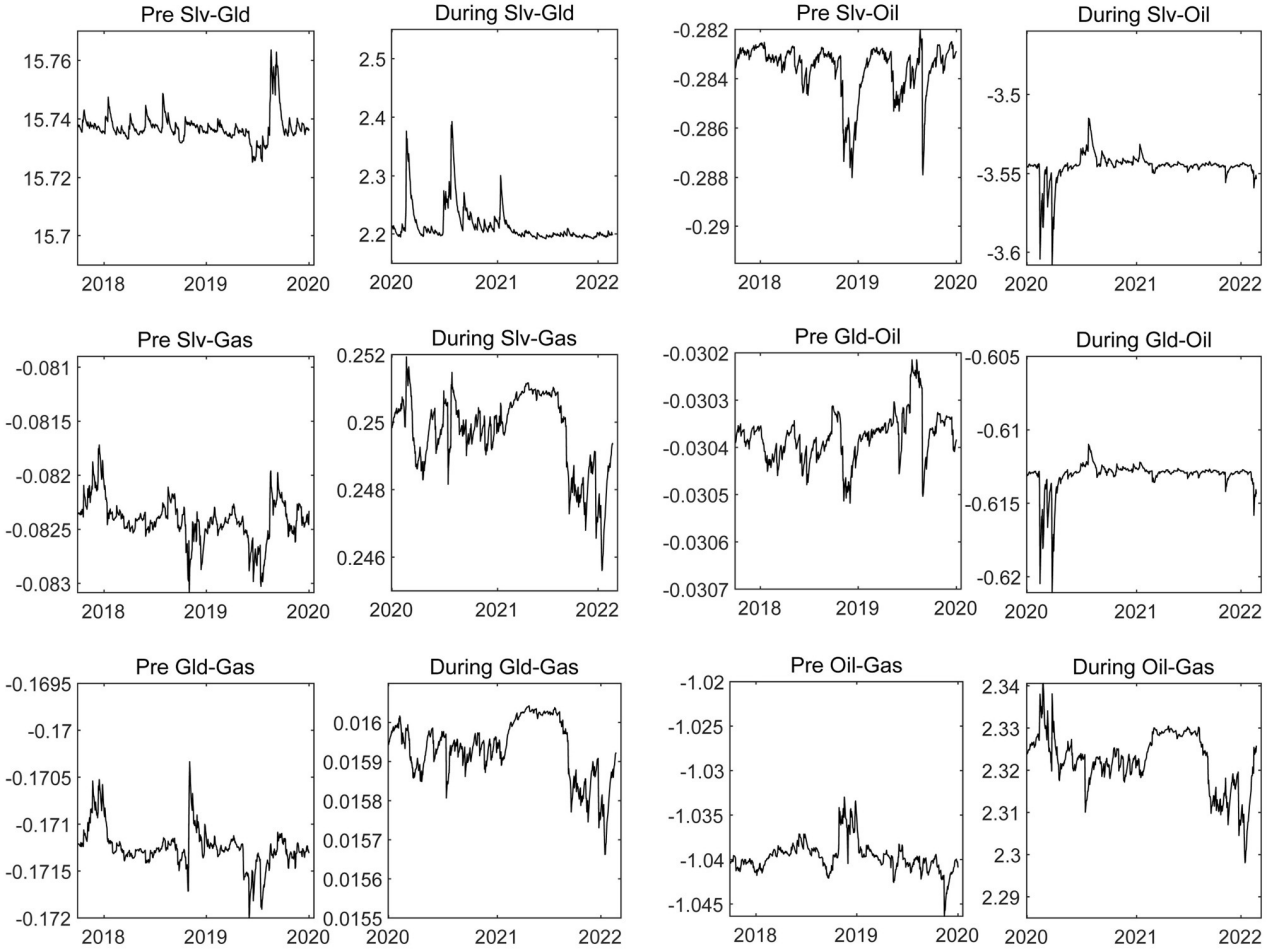
The time-varying net pairwise connectedness of energy and precious metals markets. “Pre” indicates the period before the COVID-19 epidemic, and “During” indicates the COVID-19 epidemic period, and “Slv-Gld” indicates the volatility spillover connectedness between silver market and gold market. If the volatility connectedness index is > 0, the former is the spillover and the latter is receiver of external volatility spillover. If the connectedness index is <0, the opposite is true. The results of other net pairwise connectedness are interpreted in the same way as above.

The in-depth exploration of the time-varying volatility spillover connectedness among the energy and precious metals markets was the key point for clarifying the price risk spillover path and spillover tendency among those markets amid the COVID-19 epidemic. Figure 4 shows the time-varying connectedness of volatility spillovers among the four commodity markets. There was high degree of integration within the precious metals market before the COVID-19 epidemic and a weak volatility spillover connectedness between the precious metals and energy markets. For example, before 2020, the volatility spillover between the silver and gold markets was as high as 15%, indicating a strong risk synergy between them. However, following the outbreak of COVID-19, the volatility spillover level between the precious metals markets decreased rapidly, while the risk connection between the precious metals and energy markets enhanced significantly. This indicates that the negative impact of the COVID-19 epidemic enlarged the risk absorption capacity of the precious metals market and made it the terminal point of risk transmission for other markets. This relates to its own characteristics, such as the scarcity, independence, and lack of restriction by any country or trade market.

We further found that the COVID-19 epidemic has intensified the volatility of the crude oil market. Under the shock of the epidemic, the crude oil market has become the source of risk spillover for all other markets, and the risk transmission of the crude oil market has been directional. The silver market was found to be the main transmission object, and the market risk was found to have finally been digested by the gold market, indicating that the silver market plays a major role in the risk transfer station of the gold market. It is worth noting that the outbreak of the COVID-19 epidemic enhanced the risk absorption capacity of the natural gas market and magnified its role in stabilizing the risk of price volatility. Therefore, the natural gas market has also become the main gathering place for risks during the COVID-19 epidemic and is mainly connected with the risk spillover from the crude oil market. It must be emphasized that the stability of the above risk transmission path also showed to have time-varying characteristics, and this stability improved as the epidemic became more severe. In the early stages of the COVID-19 outbreak in 2020 and in the middle and early periods of 2021, the stability of the risk connection, spillover path, and tendency among markets significantly improved, indicating that our above conclusions are robust.

## Conclusion

In this article, we utilized the DCC-GARCH-CONNECTEDNESS approach to explore the volatility spillover connectedness before and during the COVID-19 epidemic. Based on existing literature, relevant research conclusions were enriched and expanded. The main conclusions of this paper are as follows.

First, the outbreak of the COVID-19 epidemic strengthened the volatility correlations among the gold, silver, crude oil, and natural gas markets and enhanced the risk connection among them. Second, the COVID-19 epidemic has intensified the price risk of the crude oil market and increased its volatility risk spillover level. Therefore, under the shock of the epidemic, the crude oil market has transitioned from the receiver of market volatility risk to the spillover. It has also become the source of the risk transmission of commodity markets in the post-epidemic era. Third, the outbreak of the epidemic enhanced the risk reception capacity of the natural gas and silver markets, as well as amplified its role in stabilizing the risk of price volatility. It also weakened the risk absorption capacity of the gold market, though the gold market remains the terminus of external volatility risk as the risk stabilizer. In addition, the COVID-19 epidemic has weakened the volatility spillover level within the precious metals market and enhanced the spillover connection between the precious metals and energy markets, which fully highlights the risk mitigation function of the precious metals markets. Fourth, the volatility spillover level between markets showed clear time-varying characteristics and positively correlated with the severity of the COVID-19 epidemic. This means that increases in the severity of the COVID-19 epidemic will amplify the volatility spillover level of each market in the same direction.

Some policy implications can be drawn from the above conclusions. First, because the COVID-19 epidemic was shown to have intensified the risk volatility spillover among commodity markets, governments should attempt to prevent and control the epidemic, restore consumer confidence, and improve the industrial chain structure. International organizations should also play an active role. They can smooth the price volatility of financial assets through control measures such as open-market operations and maintain the stable trend of commodity prices by improving the liquidity of commodity markets. Doing so could prevent the internal risk volatility connection among commodity markets. Moreover, considering the precious metals market as the reception of risk spillover, the government should guide investors to effectively utilize the risk aversion performance of gold and silver and bring precious metals and commodities with large risk spillover into the portfolio basket to realize risk hedging. This could allow them to improve overall investment efficiency and weaken the turbulence caused by external risk shocks as well as give full play to the role of gold as a risk stabilizer in the COVID-19 epidemic. In addition, the prevention and control of crude oil market risk volatility spillover deserves greater attention. At the macro level, governments should improve commodity markets and play a positive role in the global value chain of energy and trade networks. At the micro level, they should guide arbitrage transactions, which can effectively reduce risk exposure, to enrich crude oil price risk management tools, and prevent the negative impact of speculative factors under the shadow of the epidemic. Furthermore, based on the increasingly prominent risk transmission position of the natural gas market, there is an opportunity to build the power of natural gas demand and promote the establishment of a joint bargaining mechanism, which can fundamentally weaken the risk transmission function of the natural gas market and ensure market and financial stability. Finally, as the energy market becomes a risk spillover center, governments should increase the development and utilization of renewable energy. Grasping the opportunity for energy transformation partly granted by the epidemic and actively introducing clean energy, such as solar and wind energy and their related commodities, into financial markets are also important measures. In particular, they are helpful for hedging the instability of traditional energy with the sustainability of renewable energy and for strengthening the risk prevention of international financial markets.

## Data Availability Statement

The original contributions presented in the study are included in the article/supplementary material, further inquiries can be directed to the corresponding author.

## Author Contributions

XT: conceptualization, validation, writing original draft, supervision, and funding acquisition. XW: methodology and writing—review and editing. SM: writing—review and editing and visualization. ZW: software, visualization, and formal analysis. YZ: validation, resources, and data curation. LX: software, data curation, project administration, and visualization. All authors contributed to the article and approved the submitted version.

## Funding

We acknowledge the financial support from Youth Foundation of Humanities and Social Sciences of the Ministry of Education in China (20YJC790122).

## Conflict of Interest

The authors declare that the research was conducted in the absence of any commercial or financial relationships that could be construed as a potential conflict of interest.

## Publisher's Note

All claims expressed in this article are solely those of the authors and do not necessarily represent those of their affiliated organizations, or those of the publisher, the editors and the reviewers. Any product that may be evaluated in this article, or claim that may be made by its manufacturer, is not guaranteed or endorsed by the publisher.

## References

[1] Tang W Wu L Zhang ZX. Oil price shocks and their short- and long-term effects on the Chinese economy. Energy Econ. (2010) 32:S3–14. 10.1016/j.eneco.2010.01.002

[2] Ewing BT Malik F Ozfidan O. Volatility transmission in the oil and natural gas markets. Energy Econ. (2002) 24:525–38. 10.1016/S0140-9883(02)00060-9

[3] Baur DG McDermott TK. Why is gold a safe haven? J Behav Exp Finance. (2016) 10:63–71. 10.1016/j.jbef.2016.03.002

[4] Bouoiyour J Selmi R Wohar ME. Safe havens in the face of presidential election uncertainty: a comparison between bitcoin, oil and precious metals. Appl Econ. (2019) 51:6076–88. 10.1080/00036846.2019.1645289

[5] Mensi W Sensoy A Vo XV Kang SH. Impact of COVID-19 outbreak on asymmetric multifractality of gold and oil prices. Res Policy. (2020) 69:101829. 10.1016/j.resourpol.2020.101829 34173419 PMC7420105

[6] Vacha L Barunik J. Co-movement of energy commodities revisited: evidence from wavelet coherence analysis. Energy Econ. (2012) 34:241–7. 10.1016/j.eneco.2011.10.007

[7] Elgammal MM Ahmed WMA Alshami A. Price and volatility spillovers between global equity, gold, and energy markets prior to and during the COVID-19 pandemic. Res. Policy. (2021) 74:102334. 10.1016/j.resourpol.2021.102334 34511700 PMC8418324

[8] Xia T Ji Q Zhang D Han J. Asymmetric and extreme influence of energy price changes on renewable energy stock performance. J Clean Prod. (2019) 241:118338. 10.1016/j.jclepro.2019.118338

[9] Gabauer D. Volatility impulse response analysis for DCC-GARCH models: the role of volatility transmission mechanisms. J Forecast. (2020) 39:788–96. 10.1002/for.2648

[10] Karim S Khan S Mirza N Alawi S Taghizadeh-hesary F. Climate finance in the wake of COVID-19: connectedness of clean energy with conventional energy and regional stock markets. Clim Chang Econ. (2022). 10.1142/S2010007822400085. [Epub ahead of print].

[11] Diebold FX Yilmaz K. On the network topology of variance decompositions: Measuring the connectedness of financial firms. J Econom. (2014) 182:119–34. 10.1016/j.jeconom.2014.04.012

[12] Mensi W Rehman MU Vo XV. Spillovers and co-movements between precious metals and energy markets: implications on portfolio management. Res Policy. (2020) 69:101836. 10.1016/j.resourpol.2020.101836

[13] Goodell JW. COVID-19 and finance: Agendas for future research. Finan Res Lett. (2020) 35:101512. 10.1016/j.frl.2020.101512 32562472 PMC7152896

[14] Baker SR Bloom N Davis SJ Kost KJ Sammon MC Viratyosin T. The unprecedented stock market impact of COVID-19. NBER. (2020) 26945. 10.3386/w26945

[15] Zhang D Hu M Ji Q. Financial markets under the global pandemic of COVID-19. Finance Res Lett. (2020) 36:101528. 10.1016/j.frl.2020.101528 32837360 PMC7160643

[16] Al-Awadhi AM Alsaifi K Al-Awadhi A Alhammadi S. Death and contagious infectious diseases: Impact of the COVID-19 virus on stock market returns. J Behav Exp Finance. (2020) 27:100326. 10.1016/j.jbef.2020.100326 32292707 PMC7144859

[17] Halling M. Yu J, Zechner J. How did COVID-19 affect firms' access to public capital markets? Rev Corp Finance Stud. (2020) 9:501–33. 10.1093/rcfs/cfaa008 PMC7337854 40504142

[18] Kinateder H Campbell R Choudhury T. Safe haven in GFC versus COVID-19: 100 turbulent days in the financial markets. Finance Res Lett. (2021) 43:101951. 10.1016/j.frl.2021.101951

[19] Salisu AA Ebuh GU Usman N. Revisiting oil-stock nexus during COVID-19 pandemic: Some preliminary results. Int Rev Econ Finance. (2020) 69:280–94. 10.1016/j.iref.2020.06.023

[20] Ben-Salha O Mokni K. Detrended cross-correlation analysis in quantiles between oil price and the US stock market. Energy. (2022) 242:122918. 10.1016/j.energy.2021.122918

[21] Bhatia V Das D Kumar SB. Hedging effectiveness of precious metals across frequencies: evidence from wavelet based dynamic conditional correlation analysis. Phys A Stat Mech Appl. (2020) 541:123631. 10.1016/j.physa.2019.123631

[22] Lahiani A Mefteh-Wali S Vasbieva DG. The safe-haven property of precious metal commodities in the COVID-19 era. Resour Policy. (2021) 74:102340. 10.1016/j.resourpol.2021.102340

[23] Sharif A Aloui C Yarovaya L. COVID-19 pandemic, oil prices, stock market, geopolitical risk and policy uncertainty nexus in the US economy: fresh evidence from the wavelet-based approach. Int Rev Financial Anal. (2020) 70:101496. 10.1016/j.irfa.2020.101496 PMC7227524 38620230

[24] Bouri E Demirer R Gupta R Pierdzioch C. Infectious diseases, market uncertainty and oil market volatility. Energies. (2020) 13:4090. 10.3390/en13164090

[25] Nguyen XP Hoang AT Ölçer AI. Record decline in global CO2 emissions prompted by COVID-19 pandemic and its implications on future climate change policies. Energy Sources A Recovery Util Environ Eff . (2021). 10.1080/15567036.2021.1879969. [Epub ahead of print].

[26] Belhadj H Hamad SB. Gold and Bitcoin are Safe-haven? Evidence from Developed and Emerging Market Indices During the COVID-19 Bear Market. World Scientific Publishing Co. Pte. Ltd (2021).

[27] Baffes J. Oil spills on other commodities. Res Policy. (2007) 32:126–34. 10.1596/1813-9450-43338661333

[28] Zhang H Chen J Shao L. Dynamic spillovers between energy and stock markets and their implications in the context of COVID-19. Int Rev Financial Anal. (2021) 77:101828. 10.1016/j.irfa.2021.101828 PMC9759835 36570866

[29] Si DK Li XL Xu X Fang Y. The risk spillover effect of the COVID-19 pandemic on energy sector: evidence from China. Energy Econ. (2021) 102:105498. 10.1016/j.eneco.2021.105498 34898735 PMC8652837

[30] Adekoya OB Oliyide JA. How COVID-19 drives connectedness among commodity and financial markets: evidence from TVP-VAR and causality-in-quantiles techniques. Res Policy. (2021) 70:101898. 10.1016/j.resourpol.2020.101898 34173426 PMC7572357

[31] Diebold FX Yilmaz K. Measuring financial asset return and volatility spillovers, with application to global equity markets. Econom J. (2009) 119:158–71. Available online at: https://www.sas.upenn.edu/~fdiebold/papers/paper75/DY2final.pdf

[32] Diebold FX Yilmaz K. Better to give than to receive: Predictive directional measurement of volatility spillovers. Int J Forecast. (2012) 28:57–66. 10.1016/j.ijforecast.2011.02.00634745369

[33] Ben Amar A Bélaïd F Ben Youssef A Guesmi K. Connectedness among regional financial markets in the context of the COVID-19. Appl Econ Lett. (2021) 28:1789–96. 10.1080/13504851.2020.1854434

[34] Naeem MA Sehrish S Costa MD. COVID-19 pandemic and connectedness across financial markets. Pac Account Rev. (2021) 33:165–78. 10.1108/PAR-08-2020-011434173426

[35] Farid S Kayani GM Naeem MA Shahzad SJH. Intraday volatility transmission among precious metals, energy and stocks during the COVID-19 pandemic. Res Policy. (2021) 72:102101. 10.1016/j.resourpol.2021.102101

[36] So MK P Chan LSH Chu AMY. Financial network connectedness and systemic risk during the COVID-19 Pandemic. Asia-Pac Financ Mark. (2021) 28:649–65. 10.1007/s10690-021-09340-w34955992

[37] Le TH Do HX Nguyen DK Sensoy A. Covid-19 pandemic and tail-dependency networks of financial assets. Finance Res Lett. (2021) 38:101800. 10.1016/j.frl.2020.101800 33100926 PMC7572436

[38] Corbet S Goodell JW Günay S. Co-movements and spillovers of oil and renewable firms under extreme conditions: New evidence from negative WTI prices during COVID-19. Energy Econom. (2020) 92:104978. 10.1016/j.eneco.2020.104978 33106713 PMC7577230

[39] Yousaf I. Risk transmission from the COVID-19 to metals and energy markets. Res Policy. (2021) 73:102156. 10.1016/j.resourpol.2021.102156 PMC9606204 36317126

[40] Sharma N. Forecasting oil price volatility. Virginia Tech. (1998).

[41] Narayan PK Narayan S. Modelling oil price volatility. Energy Policy. (2007) 35:6549–53. 10.1016/j.enpol.2007.07.020

[42] Engle R. Dynamic conditional correlation: a simple class of multivariate generalized autoregressive conditional heteroskedasticity models. J Bus Econ Stat. (2002) 20:339–50. 10.1198/073500102288618487

[43] Bollerslev T. Generalized autoregressive conditional heteroskedasticity. J Econom. (1986) 31:307–27. 10.1016/0304-4076(86)90063-1

[44] Hansen PR Lunde A. A forecast comparison of volatility models: does anything beat a GARCH (1, 1)? J Appl Econom. (2005) 20:873–89. 10.1002/jae.800

[45] Koop G Pesaran MH Potter SM. Impulse response analysis in nonlinear multivariate models. J Econom. (1996) 74:119–47.

[46] Pesaran HH Shin Y. Generalized impulse response analysis in linear multivariate models. Econom Lett. (1998) 58:17–29.11369086

[47] Benlagha N El Omari S. Connectedness of stock markets with gold and oil: new evidence from COVID-19 pandemic. Finance Res Lett. (2021) 47:102373. 10.1016/j.frl.2021.102373 35431671 PMC8995523

[48] Petrella L Laporta AG Merlo L. Cross-country assessment of systemic risk in the European stock market: evidence from a CoVaR analysis. Soc Indic Res. (2019) 146:169–86. 10.1007/s11205-018-1881-8

[49] Xiang LJ Ma SQ Yu L Wang WH Yin ZC. Modelling the global dynamic contagion of COVID-19. Front Public Health. (2022) 9:809987. 10.3389/fpubh.2021.809987 35096753 PMC8795671

[50] Junttila J Pesonen J Raatikainen J. Commodity market based hedging against stock market risk in times of financial crisis: The case of crude oil and gold. J Int Finan Market Institut Money. (2018) 56:255–80. 10.1016/j.intfin.2018.01.002

[51] Mensi W Al-Yahyaee KH Kang SH. Time-varying volatility spillovers between stock and precious metal markets with portfolio implications. Res Policy. (2017) 53:88–102. 10.1016/j.resourpol.2017.06.001

